# Mitochondrial genomes of Macropsini (Hemiptera: Cicadellidae: Eurymelinae): Structural features, codon usage patterns, and phylogenetic implications

**DOI:** 10.1002/ece3.70268

**Published:** 2024-09-10

**Authors:** Meishu Guo, JiaJia Wang, Hu Li, Kai Yu, Yanqiong Yang, Min Li, Guy Smagghe, RenHuai Dai

**Affiliations:** ^1^ Guizhou Provincial Key Laboratory for Agricultural Pest Management of the Mountainous Region, Institute of Entomology Guizhou University Guiyang P. R. China; ^2^ College of Biology and Food Engineering Chuzhou University Chuzhou P. R. China; ^3^ Shaanxi Key Laboratory of Bio‐Resources Shaanxi University of Technology Hanzhong P. R. China; ^4^ Department of Plants and Crops Ghent University Ghent Belgium; ^5^ Department of Biology Vrije Universiteit Brussels (VUB) Brussels Belgium

**Keywords:** codon usage bias, Macropsini, mutation pressure, natural selection, phylogeny

## Abstract

Macropsini is a tribe of Eurymelinae in the family Cicadellidae that is widely distributed worldwide. Still, its taxonomic status has been unstable, and the classification of certain clades at the genus level has been controversial. The aim of this study is to address the patterns and processes that explain the structure and the evolution of the mitogenomes of Macropsini, while contributing to the resolution of systematic issues involving five of their genera. To this task, the mitogenomes of 26 species of the tribe were sequenced and characterized, and their phylogenetic relationships were reconstructed. The results revealed that the nucleotide composition of mitochondrial genes in these 26 species was significantly skewed toward A and T. Codons ending with T or A in relative synonymous codon usage were significantly more prevalent than those ending with C or G. The parity plot, neutrality plot, and correspondence analysis revealed that mutation and selective pressure affect codon usage patterns. In the phylogenetic relationships of the Macropsini, the monophyly of *Pedionis* and *Macropsis* was well‐supported. Meanwhile, *Oncopsis* revealed paraphyletic regarding *Pediopsoides*. In conclusion, this research not only contributes the valuable data to the understanding of the mitogenome of the Macropsini but also provides a reference for future investigations on codon usage patterns, potential adaptive evolution, and the phylogeny of the mitogenome within the subfamily Eurymelinae.

## Graphical abstract

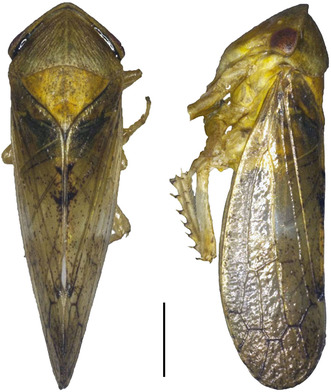

## 1 INTRODUCTION

The mitochondrial genome has been widely used in molecular systematics and population genetics due to its moderate length, relatively conserved gene arrangement, and rapid evolutionary rate (Ballard & Whitlock, 2004). Currently, with the popularization of high‐throughput sequencing technology, the number of studies comparing the mitogenomes of different insect taxa has gradually increased. These studies mainly concentrate on genome structure, base composition bias, and codon usage bias (Cameron, 2013; Jiang et al., 2019; Yang et al., 2023).

Codon usage bias (CUB) refers to the phenomenon in which synonymous codons of a species or a gene are used at different frequencies (Hasegawa et al., 1979). The study of CUB is beneficial for exploring genetic evolution and understanding gene expression characteristics. The evolution of CUB is a complex and debated issue. The mutation‐selection‐drift equilibrium model is the most widely recognized theory (Duret & Mouchiroud, 1999; Li & Tzagoloff, 1979). However, the impact of these evolutionary forces on different species remains undefined (Hershberg & Petrov, 2008). Additionally, various biological factors associated with CUB have been identified, such as base composition characteristics, GC content, gene expression level, gene length, tRNA abundance, and amino acid properties (hydrophobicity and hydrophilicity) (Min & Hickey, 2007; Yadav & Swati, 2012).

**FIGURE 1:**
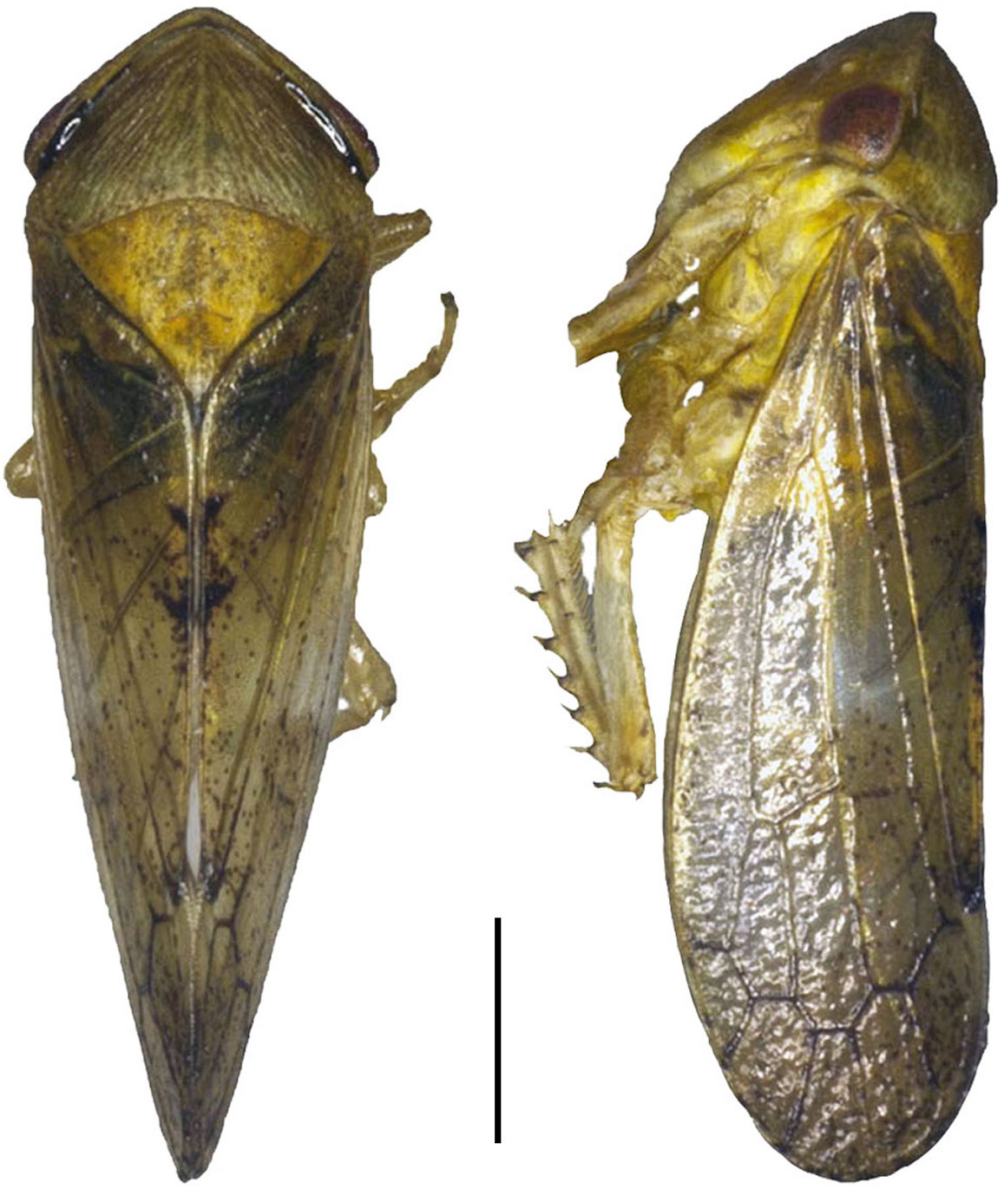
Dorsal and lateral views of *Macropsis huangbana* Li & Tishechkin.

Macropsini (Hemiptera, Auchenorrhyncha, Membracoidea, Cicadellidae, and Eurymelinae) is a tribe of Eurymelinae in the family Cicadellidae. Macropsini is mainly characterized by having the crown very broad, short, and protruding forward. The pronotum is strongly convex, covered with wrinkles and dots (Figure 1). These minute insects, colloquially known as hoppers, are plant feeders that suck plant sap from grass, shrubs, or trees. They undergo a partial metamorphosis, and interestingly, have various host associations, varying from very generalized to very specific. The insects of Macropsini are widespread worldwide, with over 750 species reported in 19 genera globally. In China, there are records of 138 species belonging to eight genera (Li, Dai, & Webb, 2023; Li, Li, et al., 2023; Li, Wang, et al., 2023; Wang, Wu, Yang, & Dai, 2020). These insects are significant ecological and commercial forest pests that mainly feed on woody plants, causing direct damage to plants by sucking plant juice and oviposition (Li et al., 2014). In addition, some species are potential pests due to their ability to spread plant viruses, such as the *Oncopsis alni* (Schrank), a vector for the grapevine yellows (Beirne, 1954; Kunkel, 1935; Maixner & Reinert, 1999).

The taxonomic history of Macropsini has seen several changes. Evans initially established Macropsidae with *Macropsis* Lewis as the type genus (Evans, 1936) and later reduced it to Macropsinae, which included Macropsini and Nioniini (Evans, 1946). Nioniini was subsequently excluded, leaving only one tribe in the subfamily Macropsinae (Linnavuori, 1978). Dietrich and Thomas (2018) further downgraded it to Macropsini and placed it and Idiocerini in the subfamily Eurymelinae. However, there are still significant uncertainties in the genus‐level classification of *Macropsis*, *Pedionis*, and *Pediopsoides*. To address this, various researchers have conducted systematic studies. For instance, Li and Dai (2018) analyzed 22 Macropsini species based on the *COX1* gene, supporting the monophyly of *Oncopis* and *Pedionis* but not of *Macropsis* and *Pediopsoides*. Subsequently, Xue et al. (2020) conducted a systematic study of Macropsini based on molecular fragments and morphological characteristics, supporting the monophyly of *Macropsis*, but leaving the taxonomic status of *Pediopsoides* unclear.

At present, there have been few studies on the complete mitogenome of the Macropsini, and only two whole mitogenome sequences are available on NCBI (https://blast.ncbi.nlm.nih.gov/Blast.cgi). In this study, the mitogenomes of 26 species of the Macropsini were sequenced using high‐throughput sequencing technology and analyzed. We combined different strategies to explore the characteristics of codon usage bias in the mitogenome of the Macropsini, providing a reference for a deeper understanding of their evolution. In addition, this study reconstructed the phylogenetic relationship among genera and species of the Macropsini using mitogenome sequences, which provides a molecular solid information base for understanding its evolution, as well as to subside further studies involving the population genetics and the evolution of Eurymelinae.

## 2 MATERIALS AND METHODS

### 2.1 Sample collection and DNA extraction

Adult samples were collected in the field using the daytime sweep net method and the nighttime light trapping method, and they were preserved in anhydrous ethanol. The samples were returned to the laboratory in a −20°C freezer until DNA extraction. The adult specimens were identified according to their morphological traits (Li et al., 2019; Li, Dai, & Webb, 2023; Li, Li, et al., 2023). Genomic DNA was extracted from a single male adult's head and chest muscle tissue using the DNeasy® Blood & Tissue Kit according to the manufacturer's recommendations. The extracted genomic DNA was stored at −20°C for further analysis. DNA samples and voucher specimens with male external genitalia were preserved at the Institute of Entomology, Guizhou University, Guiyang, China (GUGC).

### 2.2 Sequence assembly and annotation

Whole genomes for 26 species samples were sequenced using Illumina sequencing technology (Illumina HiSeq 4000 platform, 150 bp bipartite sequencing reads with an average insert size of 350 bp and 2 GB clean data; Berry Genetics, Beijing, China). The sequences from the NGS data were mapped in Geneious v 2019.2.1 using the “Map to Reference” function with a Medium‐Low sensitivity and five times iteration, using 600 bp *COI* sequences of *Macropsis notata* (GenBank NC_042723) and *Oncopsis nigrofasciata* (GenBank MG_813492) (Wang, Wu, Yang, & Dai, 2020) as references. After that, the previously acquired results served as a fresh reference sequence, and the assembly procedure described above was repeated until all the mitogenomic reads were extracted. The retrieved mitogenome sequence was preliminary annotated using the MITOS web server (http://mitos.bioinf.uni‐leipzig.de/index.py) (Bernt et al., 2012) based on the mitochondrial genetic code of invertebrates. The NCBI ORF Finder function was then used to locate 13 protein‐coding genes (PCGs) based on the mitochondrial genetic code of invertebrates, and the abnormal initiation and termination codons were determined by comparing and correcting with previously published mitochondrial PCGs from related species (Wang et al., 2017). ARWEN v.1.2 (Laslett & Canbäck, 2007) and tRNAscan‐SE v.1.21 (Lowe & Eddy, 1997) were used to localize 22 tRNA genes. The location of the neighboring tRNA gene was used to pinpoint the location of the rRNA genes, which were identified by comparison with genes of other Hemipteran insects. Finally, the assembled sequences were uploaded to NCBI (https://www.ncbi.nlm.nih.gov/), and the accession numbers are shown in Table 1.

**TABLE 1:** Accession numbers in NCBI of 26 species of the Macropsini.

Genus	Species	Accession number	Genus	Species	Accession number
*Oncopsis*	*Oncopsis* sp.	OR637849	*Macropsis*	*Macropsis longiprocessa*	OR637861
	*Oncopsis serrulota*	OR637848		*Macropsis flavida*	OR637845
	*Oncopsis odontoidea*	OR637847		*Macropsis irenae*	NC072988
	*Oncopsis nigromaculata*	OR637854		*Macropsis huangbana*	NC072989
	*Oncopsis konkaensis*	OR637855		*Macropsis matsumurana*	OR637846
	*Oncopsis fumosa*	OR637856		*Macropsis costalis*	OR637844
	*Oncopsis bimaculiformis*	OR637857		*Macropsis hainanensis*	NC072990
	*Oncopsis beishanensis*	OR637858		*Macropsis ocellata*	NC072992
	*Oncopsis anchorous*	OR637859		*Macropsis perpetua*	NC072991
*Pediopsoides*	*Pediopsoides dilata*	OR637862	*Pedionis*	*Pedionis nigrocorporis*	OR637853
	*Pediopsoides aomians*	OR637852		*Pedionis acerosa*	OR637850
*Pediopsis*	*Pediopsis* sp.	OR637851		*Pedionis papillata*	NC072993
*Macropsis*	*Macropsis rubrosternalis*	OR637860		*Pedionis sagittata*	NC072994

### 2.3 Nucleotide composition and diversity analysis

The sequences were analyzed using MEGA X (Kumar et al., 2018), regarding the percentage of overall nucleotide composition of each mitogenome (A%, T%, C%, G%), the nucleotide composition of the third position of the codons (A3%, T3%, C3%, G3%), the percentage of the GC content (G + C), the percentage of the AT content (A + T), and the frequency of the G + C of codon first and second nucleotides mean value (GC12). Strand asymmetry was calculated using the following formulas: GC skew = (G − C)/(G + C), and AT skew = (A − T)/(A + T) (Perna & Kocher, 1995).

The polymorphic sites and nucleotide diversity (Pi) of each PCG among species were determined using DnaSP v5.0 (Librado & Rozas, 2009). A sliding window of 200 bp (in 20 bp overlap steps) was implemented to calculate Pi between PCGs and rRNA genes in the alignment of 26 mitogenomes (Yang et al., 2023).

### 2.4 Codon usage

The relative synonymous codon usage (RSCU) of 13 PCGs in the mitogenome was analyzed using MEGA X (Kumar et al., 2018). A heatmap illustrating the RSCU values of the PCGs, excluding stop codons, in the 26 newly sequenced mitogenomes was plotted using Chiplot (https://www.chiplot.online/). RSCU, which can more accurately reflect the level of CUB, is the ratio of the actual observed value to the predicted value for a specific synonymous codon, regardless of gene length and amino acid frequency (Sharp & Li, 1986). An RSCU value of 1 indicates unbiased codon usage; values less than 1 suggest low preference, while values greater than 1 indicate high preference (Behura & Severson, 2012). Additionally, codons presenting RSCU values exceeding 1.6 are considered overrepresented, whereas codons presenting RSCU lower than 0.6 are considered underrepresented (Deb et al., 2021).

### 2.5 Parity Rule2 (PR2) bias plot

PR2 bias plot analysis was performed with AT‐bias = A3/(A3 + T3) as abscissa and GC‐bias = G3/(G3 + C3) as ordinate to explore the effects of mutation pressure and natural selection pressure. Theoretically, the use of A/T or G/C at the third codon position is considered proportional when the gene is simply influenced by base composition (Kawabe & Miyashita, 2003). In contrast, selective pressure and mutation pressure combined can result in a different frequency of use of A/T or G/C (Sueoka, 1995). The central position of the plot (0.5, 0.5) indicates that A = T and G = C, demonstrating that CUB is unaffected by base mutations and natural selection (Yengkhom et al., 2019).

### 2.6 Neutrality plot

Neutrality plot analysis is a method for quantitatively analyzing the influence of directed mutation pressure and natural selection on CUB. Here, we constructed the neutral plot with GC12 as the y‐axis and GC3 as the x‐axis. The correlation between GC12 and GC3 was analyzed using SPSS v26.0 software based on the Pearson correlation coefficient. When the slope of the regression curve goes to 0 and there is no significant correlation between GC12 and GC3, it is entirely influenced by natural selection. In contrast, when the slope is close to or equal to 1 and the correlation is significant, it is mainly affected by mutation pressure (Sueoka, 1988).

### 2.7 Correspondence analysis (COA)

Correlation analysis (COA) is the use of multivariate statistical methods to explore the relationship between the variables. It was used here to address the main mechanisms affecting the codon usage patterns of 13 PCGs (Perrière & Thioulouse, 2002; Shields & Sharp, 1987). The RSCU frequency of 13 PCGs was analyzed by Past 4.09 software to investigate the specific causes of CUB further. All zero‐row and stop codons (UAA and UAG) in the matrix were deleted.

### 2.8 Grand average of hydropathy (GRAVY)

The amino acid composition of 13 PCGs from 26 species was analyzed by MRGA X (Kumar et al., 2018). The Grand average of hydropathy (GRAVY) value of 13 PCGs was calculated by Galaxy (https://galaxyproject.org) (Jalili et al., 2020). The GRAVY value is typically the product of the frequency of amino acids and the corresponding hydrophobic index, which determines the hydrophobicity (positive GRAVY value) and hydrophilicity (negative GRAVY value) of proteins (Kyte & Doolittle, 1982).

### 2.9 Phylogenetic analysis

In this study, the mitogenomes of 28 species of Macropsini were selected as the ingroup, including the new 26 mitogenome sequences reported here as well as the mitogenomes of *M. notata* (GenBank NC_042723) and *O. nigrofasciata* (GenBank MG_813492) previously characterized by Wang, Wu, Yang, and Dai (2020). Two closely related species, *Gessius rufidorsus* (MN_577633) of the subfamily Iassinae and *Olidiana obliquea* (MN_780583) of the subfamily Coelidiinae, were selected as outgroups to construct the phylogenetic tree following the previous studies (Wang, Wu, Dai, & Yang, 2020). PCGs and rRNAs were extracted from the mitogenome using Geneious Prime 2019.2.1 software (Kearse et al., 2012). The MAFFT plugin in PhyloSuite v1.2.1 (Zhang et al., 2020) was used to align the set of sequences retrieved for each gene, based on the Q‐INS‐i and G‐INS‐i strategies (for PCGs and rRNAs, respectively). The well‐aligned sequences were then concatenated using MEGA X (Kumar et al., 2018) to generate separate datasets, including (1) Amino acid sequences of 13 PCGs (AA), totaling 3615 amino acids; (2) first and second codons of 13 PCGs combined with two rRNA genes (PCG12‐rRNA), totaling 9120 nucleotides; and (3) 13 PCGs combined with two rRNA genes (PCG‐rRNA), totaling 12,735 nucleotides.

To assess the presence of phylogenetic information in the sequences, three datasets (AA, PCG12‐rRNA, and PCG‐rRNA) underwent saturation analysis using DAMBE v7.0.35 (Xia et al., 2003). PartitionFinder v2.1.1 (Lanfear et al., 2017) was utilized to determine the best model for each dataset, ensuring that each gene fragment had its ideal model. The phylogenetic tree was constructed using Maximum Likelihood (ML) and Bayesian Inference (BI) based on each dataset. ML phylogenetic trees were generated with IQ‐TREE v1.6.3 using the ultrafast bootstrap approximation approach (Nguyen et al., 2015), repeated 10,000 times. BI phylogenetic trees were constructed using MrBayes v3.2.6 (Huelsenbeck & Ronquist, 2001). The BI analysis used default settings to simulate four independent operations for one million generations, sampling every 1000 generations. The first 25% of samples were discarded when the average standard deviation of split frequencies reached 0.01, and the remaining samples were used to build a consensus tree and calculate the posterior probability (PP).

## 3 RESULTS

### 3.1 Nucleotide composition and diversity analysis

**FIGURE 2:**
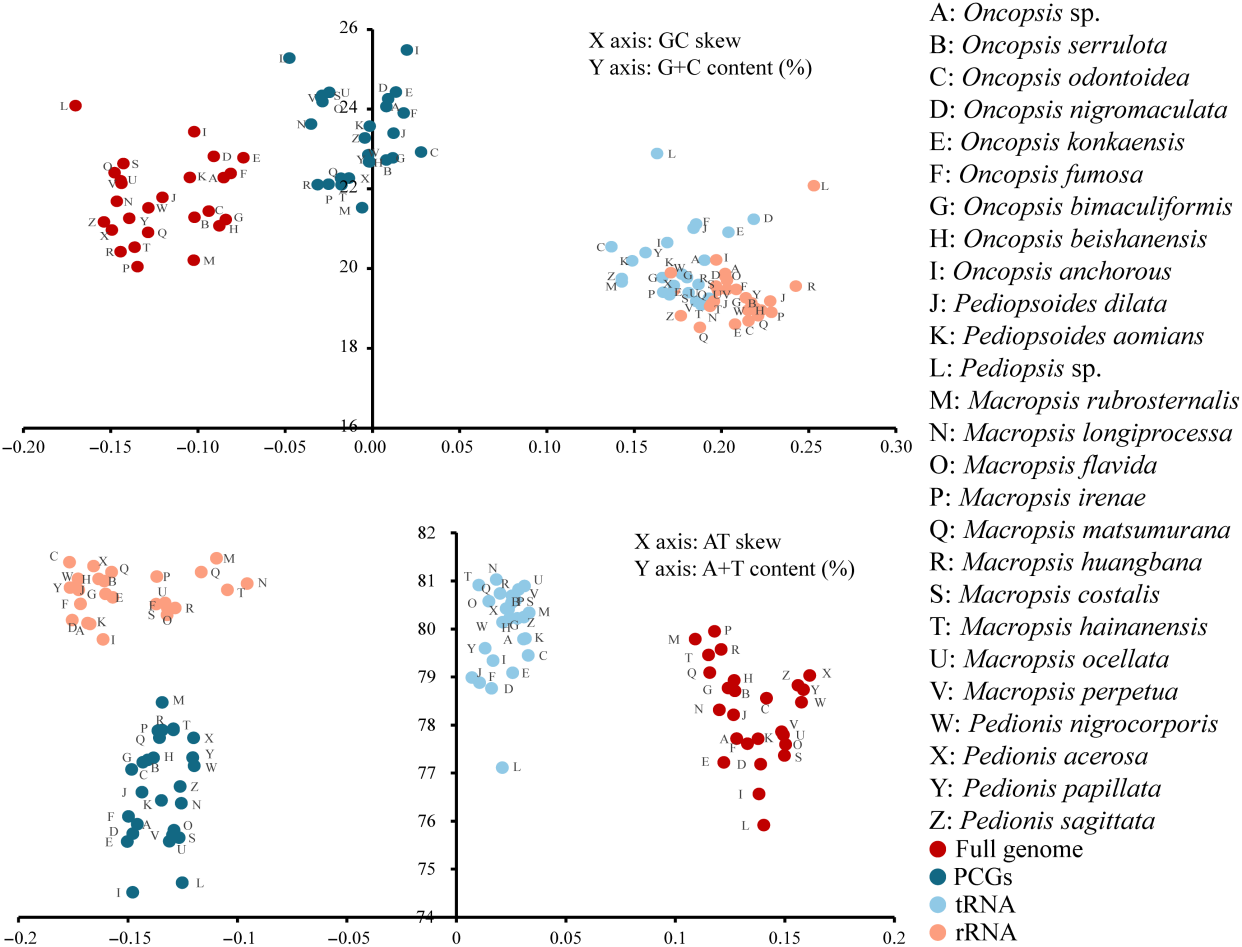
The GC skew versus G + C content (%) and AT skew versus A + T content (%) across the mitochondrial genomes of 26 species. Points are grouped in colors according to content, where each point represents a single species.

The sequence lengths of the mitogenomes from the 26 species ranged from 15,279 bp (*Pediopsis* sp.) to 16,546 bp (*Pedionis sagittata*) (Table S1). A comparative analysis revealed that the lengths of PCGs, tRNAs, and rRNAs in these mitogenomes were similar, with variations primarily observed in the control region. The nucleotide composition of 26 complete mitogenomes exhibited obvious AT bias, with A + T content ranging from 75.91% (*Pediopsis* sp.) to 79.95% (*M. irenae*), and all displayed a positive AT skew (0.109–0.161) and a negative GC skew (−0.170 to −0.074). Additionally, the A + T content of the PCGs ranged from 74.52% (*Oncopsis anchorous*) to 78.47% (*Macropsis rubrosternalis*), with a negative AT skew (−0.150 to −0.120). The A + T content of the tRNA genes ranged from 77.11% (*Pediopsis* sp.) to 81.03% (*Macropsis longiprocessa*), with all showing positive AT skew (0.007–0.033) and GC skew (0.137–0.219). The A + T content of rRNA genes ranged from 77.92% (*Pediopsis* sp.) to 81.47% (*M. rubrosternalis*), both demonstrating a negative AT skew (−0.177 to −0.096) and a positive GC skew (0.170–0.253) (Figure 2).

**FIGURE 3:**
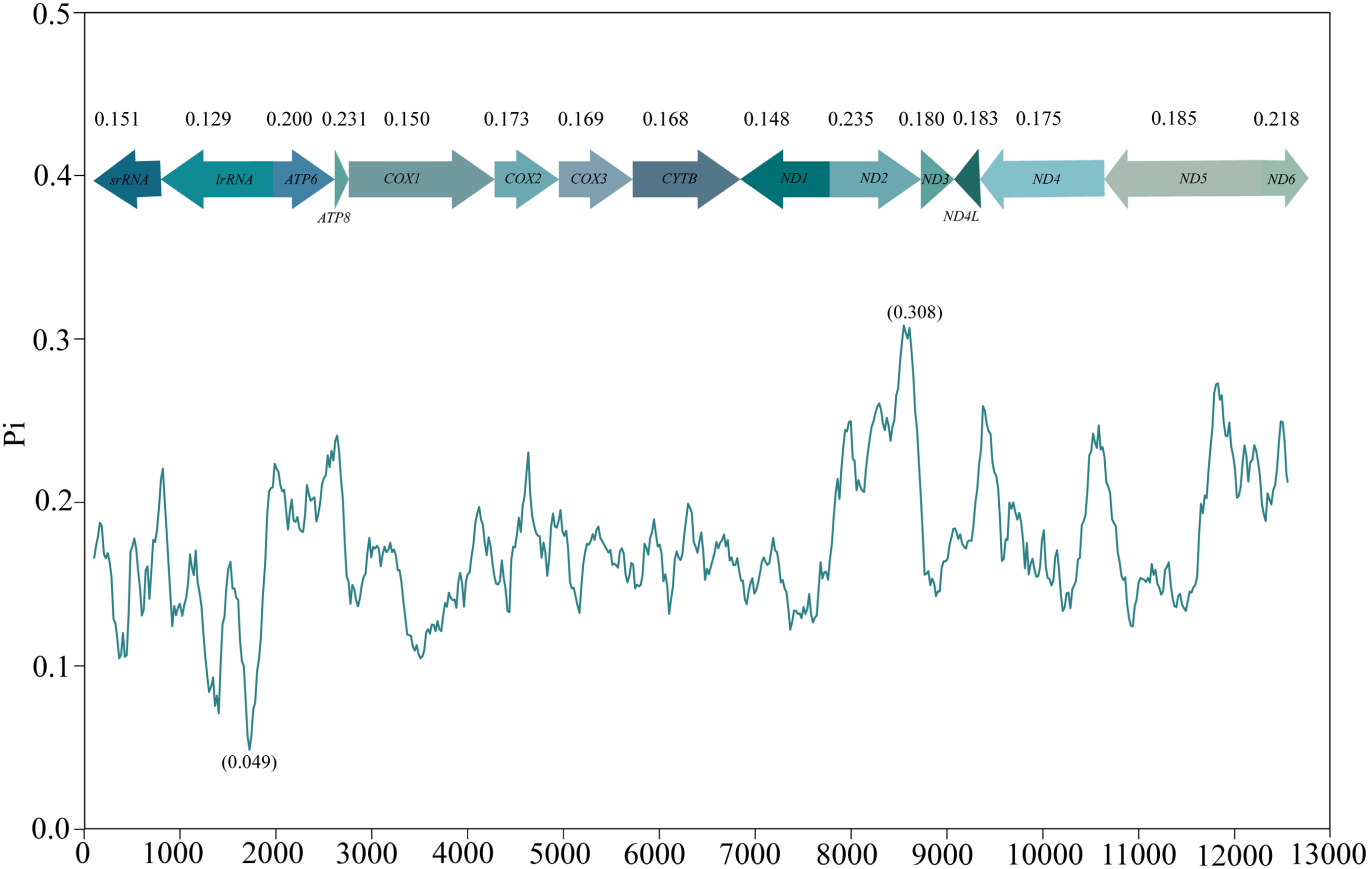
Sliding window analysis presenting distribution of Pi values across PCGs and rRNAs genes, as evaluated in mitochondrial genomes of 26 species.

Sliding‐window analysis demonstrated that Pi values (nucleotide diversity) were highly variable among the 13 PCGs and two rRNA genes in the mitogenomes of these species (Figure 3). The most polymorphic region (7714–8658 bp) was discovered in *ND2* (Pi = 0.308), and the most conserved segment (725–1890 bp) was identified in *lrRNA* (Pi = 0.049). The most variable gene was *ND2* (945 bp, Pi = 0.235), followed by *ATP8* (150 bp, Pi = 0.231), *ND6* (483 bp, Pi = 0.218), and *ATP6* (648 bp, Pi = 0.200). *COX1* (1533 bp, Pi = 0.150) and *ND1* (930 bp, Pi = 0.148) were the most conserved PCGs. The two rRNA genes were relatively conserved with *srRNA* and *lrRNA* of 0.151 and 0.129, respectively.

### 3.2 Analysis of codon usage

**FIGURE 4:**
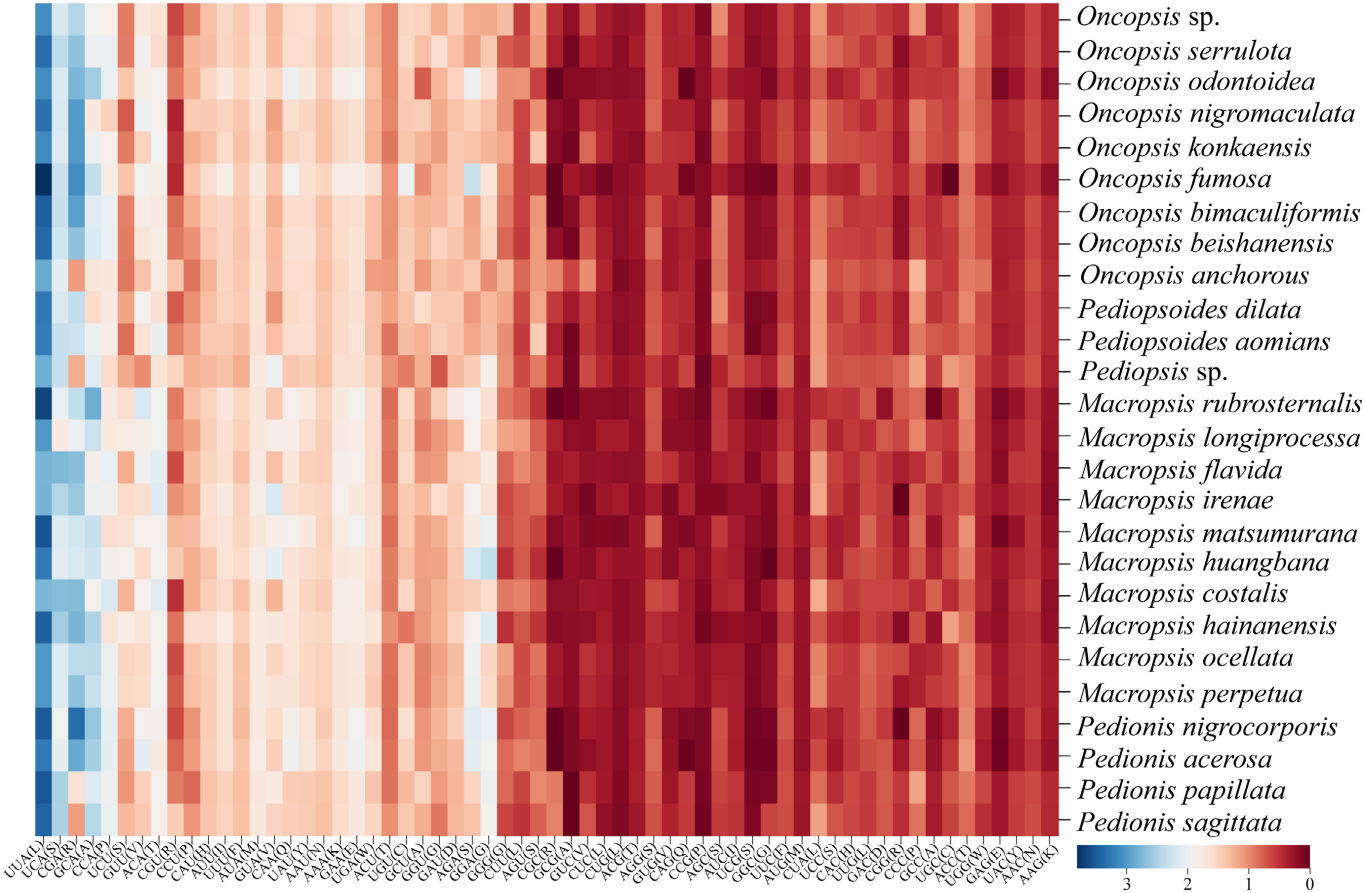
Heat map of RSCU values estimated for each codon across the mitochondrial PCGs of the 26 target species.

The RSCU values of each codon corresponding to the specific amino acids of PCGs in the mitogenomes of 26 species were examined. The findings are presented in Figure 4. It was discovered that 62 codons (aside from the two stop codons) were used unevenly in the process of coding genes, with the third position of the more frequent codons ending mostly with A or U. Thirteen codons, UUA (L), UCA (S), CCA (P), CGA (R), GCA (A), ACA (T), AUA (M), AAA (K), CAA (Q), GAA (E), GUU (V), GGA (G), and AGA (S), were overrepresented (RSCU >1.6). There were 25 underrepresented codons (RSCU <0.6), and all of them ended in G or C.

**FIGURE 5:**
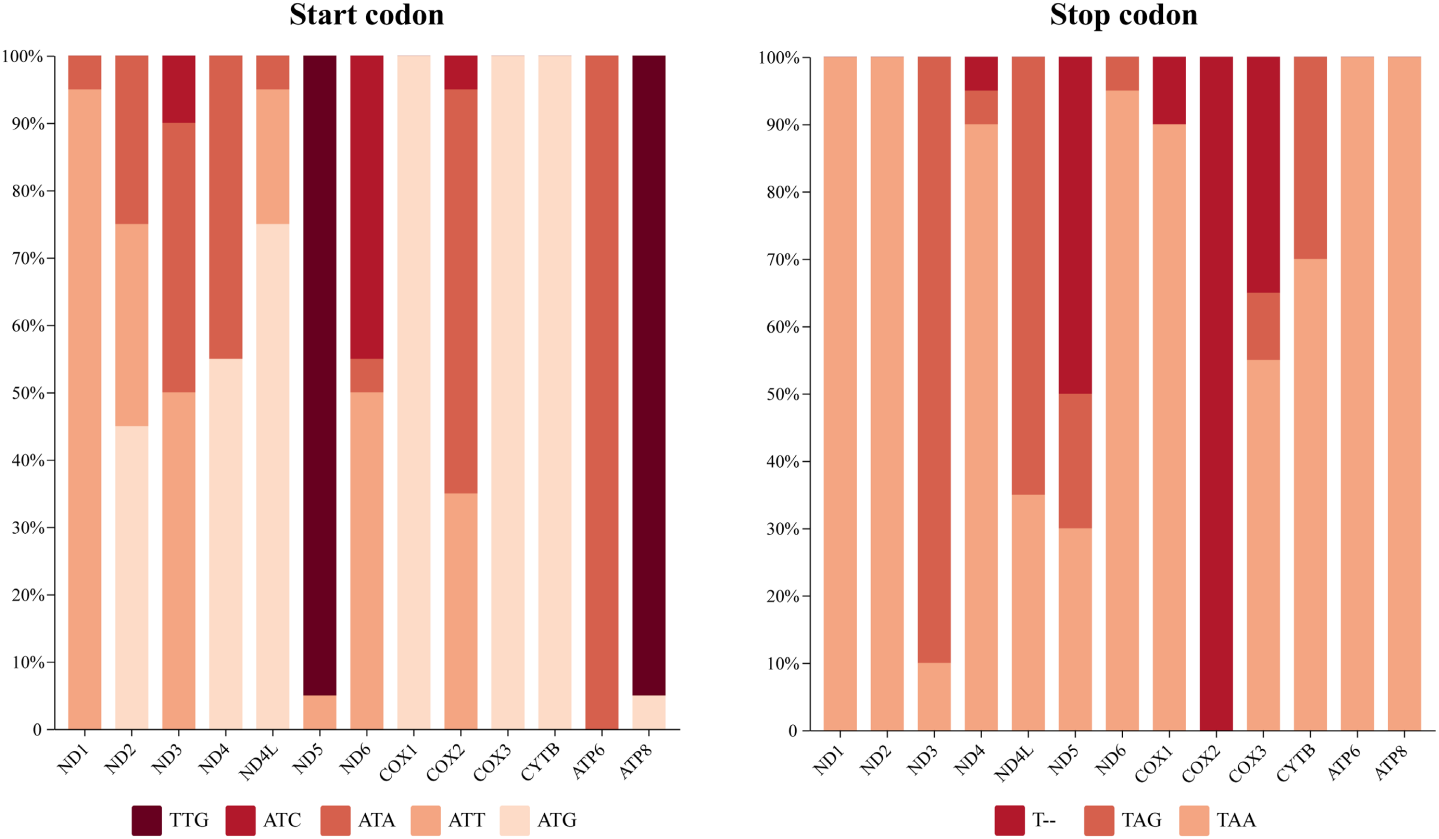
The frequency distribution of start codons and stop codons of 13 PCGs in 26 species of the Macropsini.

Except for *ND5* and *ATP8*, which started mostly with TTG, the remaining PCGs utilized the conventional start codon ATN (ATT, ATA, ATC, ATG), with ATG being the most often used initiation codon and ATC being the least frequently used. TAA, TAG, and T—were the termination codons of 13 PCGs, with TAA being the most frequently used and T—being the least frequently used (Figure 5), indicating that mitochondrial genes prefer A/T bases.

### 3.3 Parity Rule2 (PR2) bias plot analysis

**FIGURE 6:**
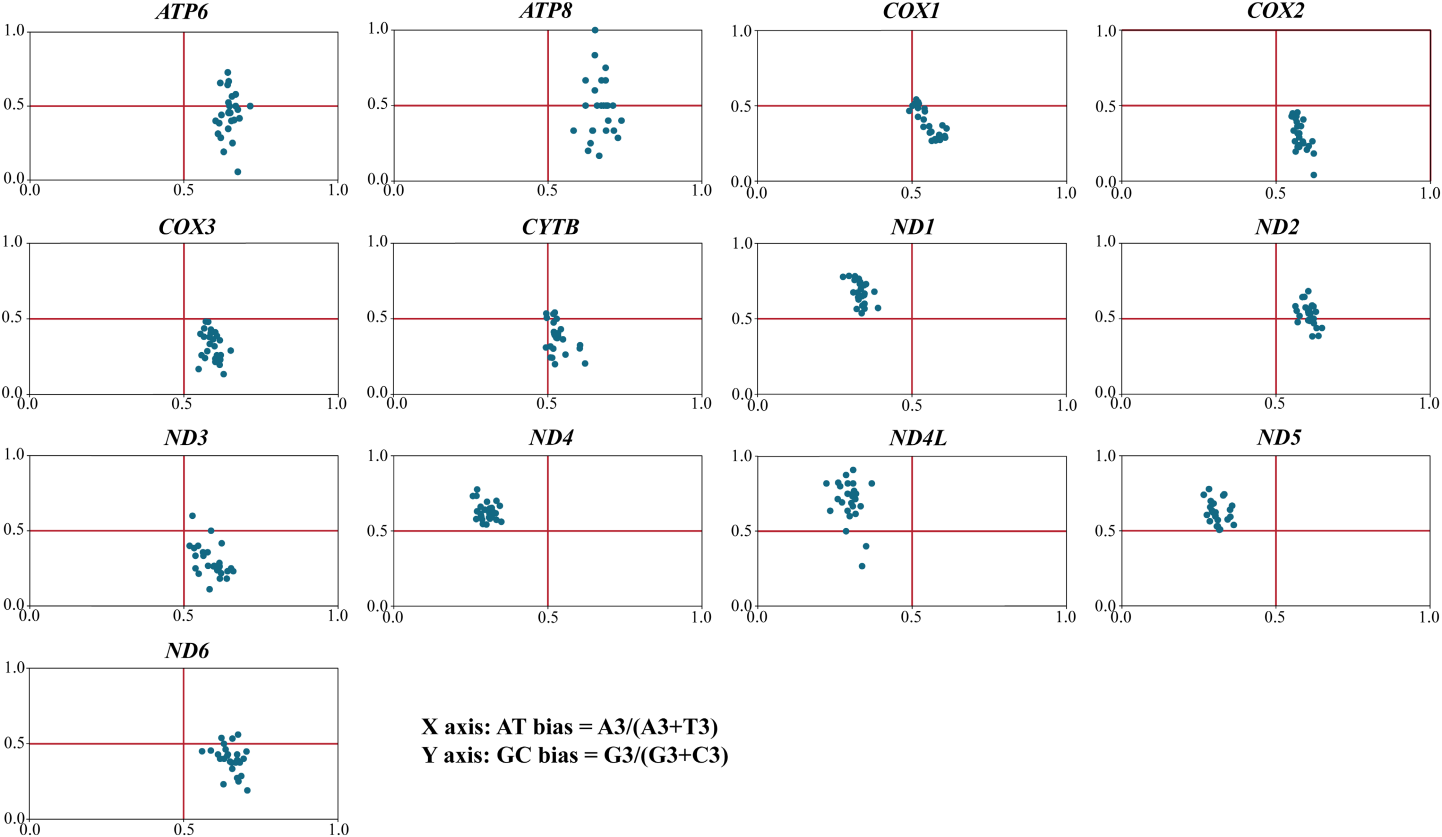
PR2‐plot analysis of the 13 mitochondrial protein‐coding genes, where each point represents one of the 26 evaluated species.

The relationship between the third bases of the codons of 13 PCGs was analyzed by Parity Rule2 bias. The results of the PR2‐plot analysis are shown in Figure 6. The genes of *ND1*, *ND4*, *ND4L*, and *ND5* were primarily dispersed unevenly in the second quadrant, with few species distributed on the median line. The frequency of the four bases in the third codon position of these genes was not consistent, showing G > C, (G3/[G3 + C3]) mean values of 0.68, 0.63, 0.70, and 0.62, respectively; T > A, (A3/[A3 + T3]) mean values of 0.33, 0.30, 0.30, and 0.31, respectively. The rest of the genes were mainly irregularly distributed in the fourth quadrant, which has a higher frequency of usage of A than T and a higher frequency of usage of C than G. It was thus evident that their codon preferences are affected by factors such as natural selection in addition to base mutations.

### 3.4 Neutrality plot analysis

**FIGURE 7:**
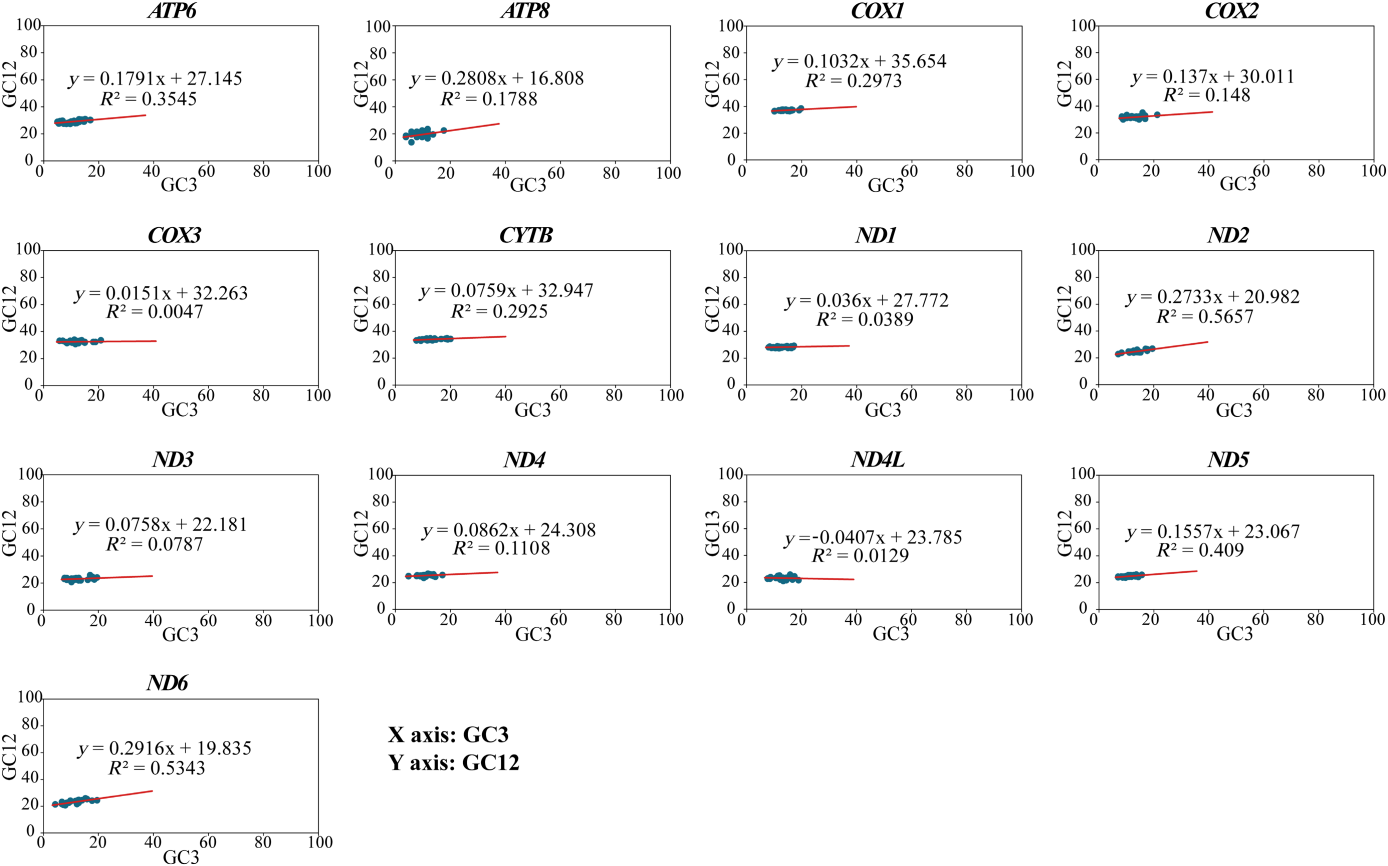
Neutrality plot analysis of 13 mitochondrial protein‐coding genes showing the correlation between GC values in the first and second x the third codon positions in the 26 evaluated species.

The results of the neutral plot analysis of the 13 PCGs are shown in Figure 7. The correlation analysis between GC12 and GC3 showed that there was a highly significant correlation (*p* < .01) between GC12 and CG3 of *ATP6*, *COX1*, *CYTB*, *ND2*, *ND5*, and *ND6* genes, as well as a moderately significant correlation for *ATP8* (*p* < .05), indicating that the mutation pattern of bases in codon positions one and two was identical to that of position three and that mutational pressure played a significant role in the CUB of these seven genes. Nevertheless, the results of the neutrality plot analysis revealed that the slopes of the regression lines of PCGs were generally lower than .5 except for the *ND2* and *ND6* genes, indicating that natural selection was an important factor influencing the CUB of PCGs. The abovementioned results indicated that the CUB of *ATP6*, *COX1*, *CYTB*, *ND5*, and *ATP8* genes were influenced by both mutation and natural selection, while *ND2* and *ND6* genes were mainly influenced by mutational pressure and other genes were mainly influenced by natural selection.

### 3.5 Correspondence analysis (COA) of codon usage

**FIGURE 8:**
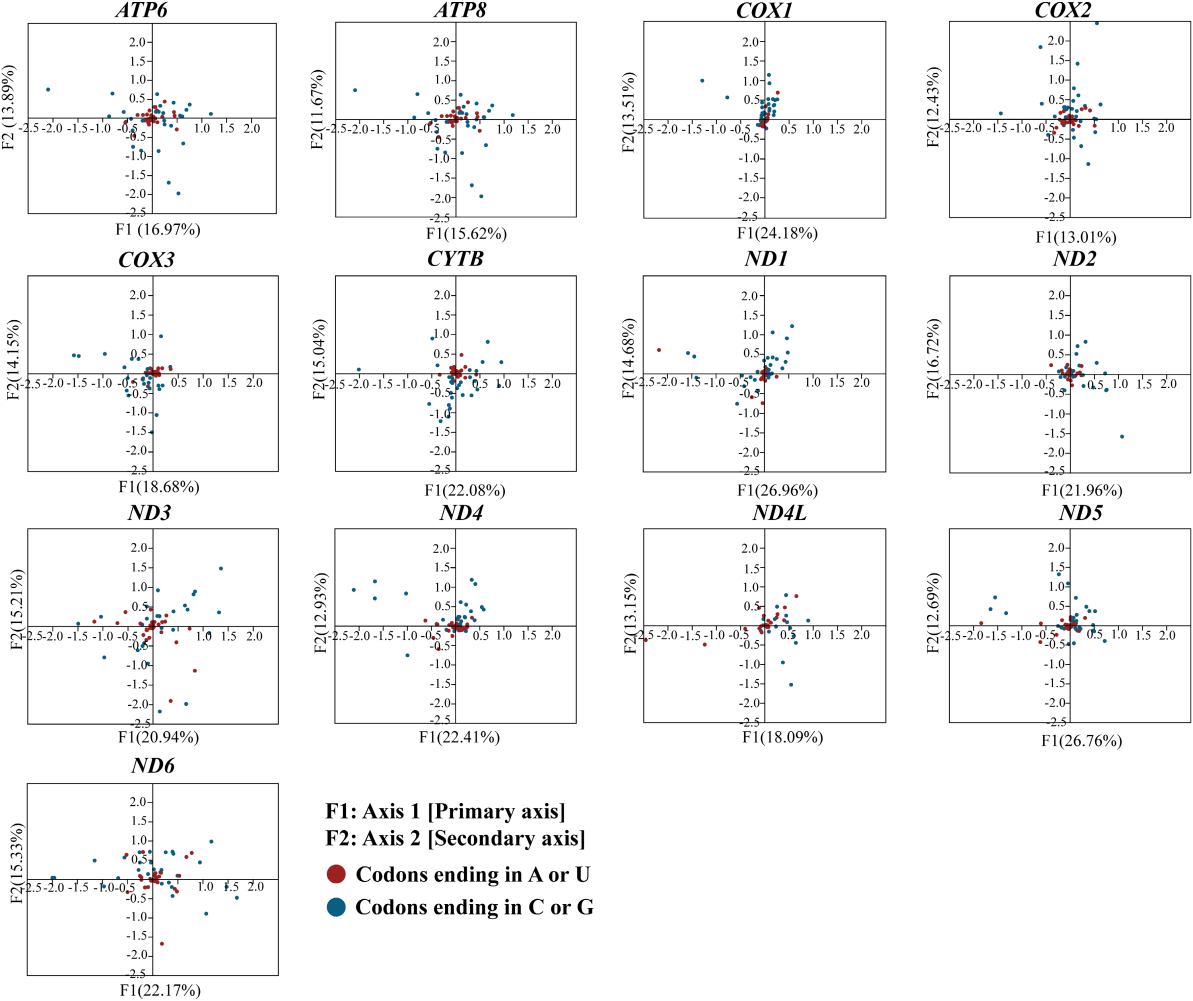
Correspondence analysis presenting the distribution of codons regarding their relative synonymous codon usage values across different protein‐coding genes of the 26 evaluated species.

Correspondence analysis is shown in Figure 8. The percentage of the variation represented by Axis 1 ranged from 13.01% (*COX2*) to 26.96% (*ND1*). The percentage of the variation represented by Axis 2 ranged from 11.67% (*ATP8*) to 16.72% (*ND2*). For several genes, some codons were discretely dispersed away from the central axis, suggesting that, besides mutation, natural selection is also influencing codon usage.

### 3.6 Analysis of amino acid composition and protein properties

**FIGURE 9:**
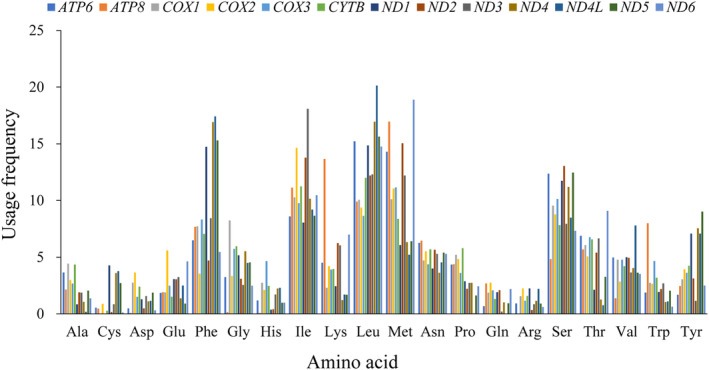
The overall frequency of amino acid usage for 13 mitochondrial protein‐coding genes in 26 species.

The total frequency of utilization of each amino acid of the 13 PCGs in the mitogenome is displayed in Figure 9. It was found that the hydrophobic amino acids Leu, Met, Ile, Phe, and Ser were most frequently used. In addition, Table 2 shows that the 13 PCGs of these species were more inclined to use hydrophobic amino acids.

**TABLE 2:** Grand average of hydropathy analysis of 13 mitochondrial protein‐coding genes in 26 species.

Species	*ATP6*	*ATP8*	*COX1*	*COX2*	*COX3*	*CYTB*	*ND1*	*ND2*	*ND3*	*ND4*	*ND4L*	*ND5*	*ND6*
*Oncopsis* sp.	0.730	0.883	0.648	0.752	0.714	0.678	0.287	0.671	0.602	0.212	0.130	0.289	0.838
*Oncopsis serrulota*	0.703	0.753	0.648	0.754	0.731	0.671	0.293	0.661	0.630	0.211	0.212	0.271	0.821
*Oncopsis odontoidea*	0.720	0.764	0.638	0.785	0.679	0.660	0.235	0.680	0.746	0.174	0.128	0.243	0.848
*Oncopsis nigromaculata*	0.689	0.739	0.636	0.788	0.722	0.690	0.299	0.718	0.621	0.217	0.175	0.247	0.848
*Oncopsis konkaensis*	0.641	0.767	0.646	0.753	0.673	0.649	0.285	0.715	0.620	0.246	0.235	0.292	0.792
*Oncopsis fumosa*	0.684	0.783	0.629	0.757	0.709	0.659	0.296	0.698	0.619	0.220	0.260	0.246	0.816
*Oncopsis bimaculiformis*	0.692	0.756	0.628	0.754	0.689	0.660	0.307	0.711	0.626	0.225	0.163	0.274	0.779
*Oncopsis beishanensis*	0.689	0.783	0.642	0.758	0.726	0.673	0.314	0.698	0.643	0.220	0.116	0.293	0.800
*Oncopsis anchorous*	0.746	0.765	0.688	0.816	0.748	0.652	0.208	0.710	0.734	0.210	0.177	0.260	0.779
*Pediopsoides dilata*	0.710	0.787	0.628	0.786	0.691	0.691	0.273	0.696	0.720	0.212	0.130	0.260	0.826
*Pediopsoides aomians*	0.724	0.769	0.664	0.783	0.684	0.682	0.259	0.782	0.659	0.226	0.173	0.278	0.846
*Pediopsis* sp.	0.773	0.912	0.759	0.847	0.805	0.770	0.274	0.753	0.808	0.222	0.106	0.294	0.721
*Macropsis rubrosternalis*	0.721	0.847	0.650	0.781	0.660	0.657	0.291	0.654	0.666	0.240	0.094	0.315	0.759
*Macropsis longiprocessa*	0.796	0.887	0.711	0.847	0.712	0.705	0.297	0.728	0.697	0.242	0.099	0.307	0.741
*Macropsis flavida*	0.787	0.907	0.742	0.819	0.762	0.733	0.237	0.728	0.712	0.202	0.151	0.265	0.828
*Macropsis irenae*	0.730	0.795	0.685	0.810	0.715	0.687	0.276	0.688	0.703	0.236	0.110	0.263	0.766
*Macropsis huangbana*	0.700	0.803	0.704	0.835	0.726	0.684	0.264	0.709	0.698	0.229	0.119	0.259	0.794
*Macropsis matsumurana*	0.751	0.768	0.653	0.807	0.704	0.679	0.276	0.670	0.673	0.253	0.137	0.292	0.760
*Macropsis costalis*	0.803	0.854	0.757	0.799	0.749	0.720	0.252	0.749	0.740	0.199	0.130	0.279	0.808
*Macropsis hainanensis*	0.731	0.812	0.684	0.801	0.689	0.687	0.324	0.678	0.705	0.221	0.078	0.301	0.771
*Macropsis ocellata*	0.788	0.871	0.756	0.817	0.760	0.716	0.249	0.724	0.705	0.206	0.118	0.258	0.823
*Macropsis perpetua*	0.787	0.871	0.756	0.826	0.746	0.718	0.254	0.727	0.715	0.213	0.122	0.264	0.815
*Pedionis nigrocorporis*	0.760	0.857	0.724	0.841	0.724	0.739	0.260	0.747	0.747	0.184	0.154	0.228	0.812
*Pedionis acerosa*	0.827	0.919	0.720	0.803	0.755	0.752	0.248	0.748	0.811	0.152	0.092	0.215	0.828
*Pedionis papillata*	0.797	0.827	0.726	0.860	0.724	0.776	0.246	0.699	0.699	0.177	0.097	0.231	0.827
*Pedionis sagittata*	0.827	0.827	0.735	0.828	0.833	0.702	0.218	0.729	0.777	0.135	0.040	0.218	0.902

### 3.7 Phylogenetic analysis

**FIGURE 10:**
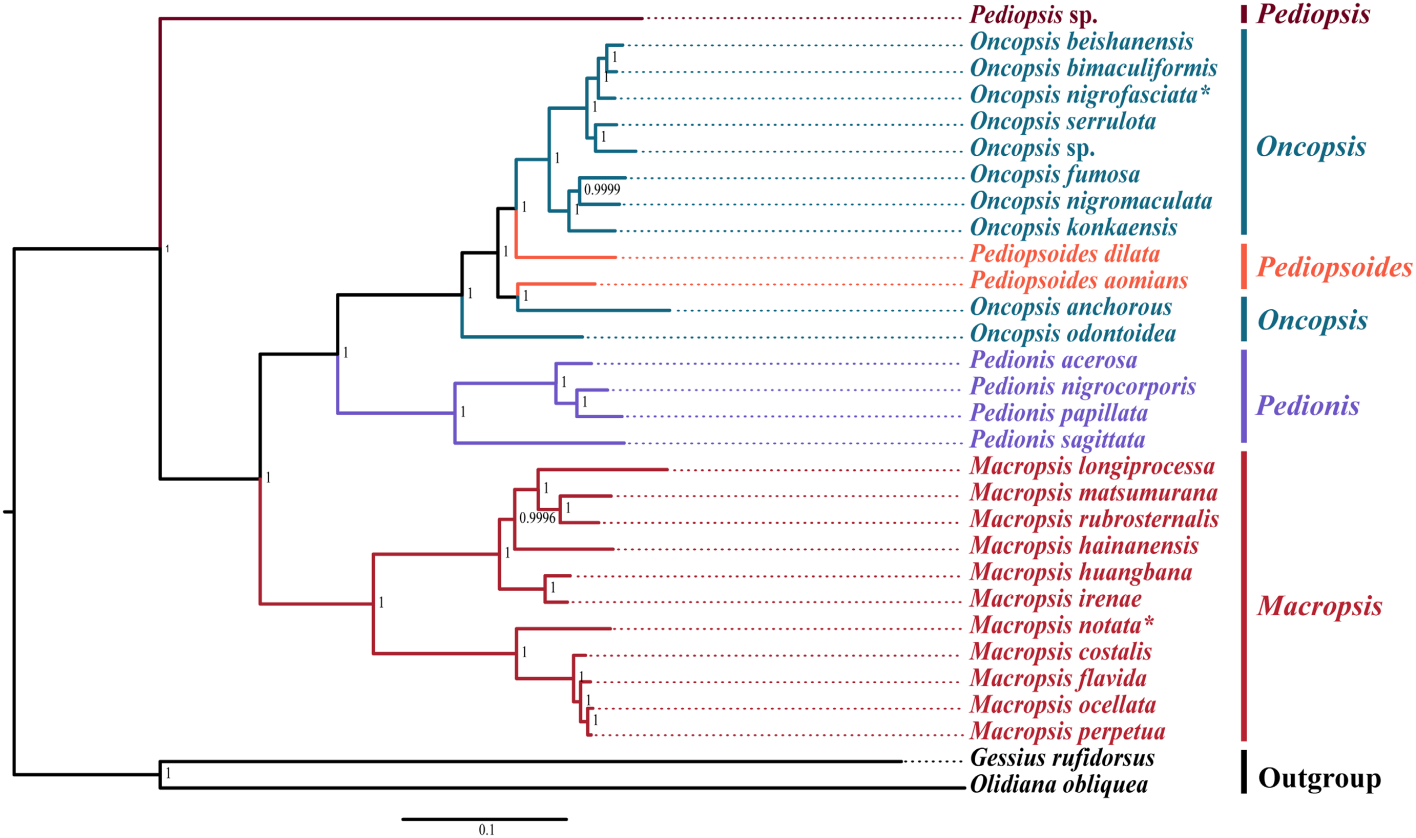
A phylogenetic tree of Macropsini was constructed using MrBayes v3.2.6 based on amino acid sequences (BI: AA). Species marked with an asterisk indicate those that have been previously published.

The substitution saturation test revealed that none of the three candidate datasets (AA, PCG12‐rRNA, and PCG‐rRNA) were saturated and that the value of the substitution saturation index (Iss) was considerably lower than the threshold value (Iss.cSym or Iss.cAsym). The two‐tailed test found that Iss significantly differed from Iss.cSym and Iss.cAsym (Table S2). This suggested that the retrieved data were suitable for further phylogenetic analyses. BI and ML studies were done on 28 species in the Macropsini using three datasets, yielding six phylogenetic trees (Figure 10; Figure S1). The results showed that the genus‐level topology of the ML and BI trees based on the mitogenome was completely consistent and most nodes received high nodal support values. In the present research, the monophyly of *Pedionis* was well supported (Bootstrap values [BS] = 100; Bayesian posterior probability [PP] = 1); the monophyly of *Macropsis* received relatively high support values in BI analyses (PP >0.88) and moderate support values in ML analyses (BS = 64–100); *Oncopsis* and *Pediopsoides* clustered into a large branch and formed a sister group relationship with *Pedionis* (BS = 100; PP = 1); *Oncopsis* revealed paraphyletic regarding *Pediopsoides*, but received relatively low support in ML analyses; *Pediopsis* was the early offshoot of Macropsini (BS = 100; PP = 1). Unfortunately, *Pediopsis* has only one representative in the dataset, making it impossible to determine its monophyly. Their phylogenetic relationships were as follows: ((((*Oncopsis* + *Pediopsoides*) + *Pedionis*) + *Macropsis*) + *Pediopsis*).

## 4 DISCUSSION

Comparative analysis indicated that the nucleotide composition of the mitochondrial genes of 26 species of the Macropsini was significantly skewed toward A and T. This finding is consistent with the previous studies of the mitogenomes of several leafhopper species, including *Macrosteles quadrimaculatus* (Du et al., 2019), *Idioscopus myrica* (Wang, Yang, et al., 2018), *Parocerus laurifoliae* (Wang, Yang, et al., 2018), and *Petalocephala gongshanensis* (Wang et al., 2022). The majority of the 13 PCGs in these 26 species utilize the typical triplet start codon ATN (ATT, ATA, ATC, ATG), whereas the termination codon TAA is most frequently used. The occurrence of this phenomenon is likely due in large part to selective avoidance of translational readthrough (TR). Previous studies have indicated that each termination codon has a different intrinsic error rate in eukaryotes, with the sequence being TGA > TAG > TAA (Geller & Rich, 1980; Parker, 1989). Therefore, it may be possible to reduce the incidence of TR by selecting for TAA (Ho & Hurst, 2022). Similarly, this study found that codons encoding the same amino acids are not used equally, and codons ending in T or A are significantly more frequent than those ending in C or G, consistent with previous findings (Wei et al., 2014).

Mutation and selection pressure effects play a significant role in the formation of codon usage patterns (Sharp et al., 2010; Wang, Meng, & Wei, 2018). A combination of PR2‐plot, neutral plot, and COA analysis indicated that the codon usage patterns in the mitogenomes of these 26 species may be impacted by a mixture of mutation and natural selection. PR2‐plot analysis revealed inconsistent frequency of the four bases in the third position, suggesting that mutation and natural selection may influence codon usage in these genes. Neutral plot analysis indicated that codon usage of *ATP6*, *COX1*, *CYTB*, *ND5*, and *ATP8* genes is influenced by both mutation and natural selection. COA analysis indicated that besides mutation, natural selection is also influencing codon usage. The exact mechanisms behind these phenomena are not fully understood, but it is evident that a discernible equilibrium exists between natural selection factors (such as gene length, gene function, and translational selection) and mutational pressures (including base content and mutation location) during the evolution of codon usage patterns (Wang et al., 2018). Additionally, we found that the majority of amino acids in the mitochondrial genomes of these 26 species are hydrophobic. Previous studies have shown that hydrophobic interaction plays a particularly dominant role in the stability of native structures (Kauzmann, 1959). It is well known that the hydrophobicity of amino acid residues plays an important role in protein folding, and it has been reported that local hydrophobicity has a greater impact on the formation of β‐sheets than on α‐helices (Kanehisa & Tsong, 1980), with codons overrepresented in β‐sheets being underrepresented in α‐helices (Das et al., 2006).

The appropriate sequencing and characterization of the target set of mitogenomes also increases the resolution of the phylogenetic relationships within Macropsini. The phylogenetic relationships, based on the three datasets in this study, supported the monophyly of both *Pedionis* and *Macropsis*. This result not only supported the monophyly of *Pedionis* but also provisionally resolved the monophyly of *Macropsis*, which was previously recovered as paraphyletic by Li and Dai (2018). *Macropsis*, the largest genus within Macropsini encompasses species with highly similar morphological features, especially genitalia (Li et al., 2012, 2014). These features can be distinguished from those of other genera, further supporting the monophyly of *Macropsis*. In addition, the phylogenetic relationships in this study indicated that *Oncopsis* revealed paraphyletic regarding *Pediopsoides*. Xue et al. (2020) conducted a study based on molecular fragments (*28S D2*, *16S*, *COX1*, *H2A*, *H3*) and 86 morphological characters, which also found that *Pediopsoides* forms a paraphyletic group, and that *Oncopsis* shares a closer relationship with *Pediopsoides*. Moreover, while observing their morphological characteristics, it was noted that some species within the *Pediopsoides* exhibit broader faces and nearly horizontal distribution marks on the pronotum. Therefore, resolving the intricate relationship between *Oncopsis* and *Pediopsoides* may require the addition or substitution of molecular markers, or a reconsideration of their taxonomic status. Dietrich et al. (2017) and Dietrich and Thomas (2018), based on morphological characteristics of leafhoppers in fossils and molecular systematic studies, classified Macropsinae and Idiocerinae into Eurymelinae as Macropsini and Idiocerini, respectively. However, the systematic studies based on mitochondrial genomes have revealed that the phylogenetic relationship between Macropsini and Idiocerini is distant (Li, Wang, et al., 2023; Wang, Wu, Dai, & Yang, 2020; Wang, Wu, Yang, & Dai, 2020), and further validation of their taxonomic status is needed in future studies.

## AUTHOR CONTRIBUTIONS

**Meishu Guo:** Data curation (lead); formal analysis (equal); methodology (lead); writing – original draft (equal); writing – review and editing (equal). **JiaJia Wang:** Conceptualization (equal); methodology (equal); resources (equal). **Hu Li:** Conceptualization (equal); methodology (equal). **Kai Yu:** Conceptualization (equal); methodology (equal); resources (equal). **Yanqiong Yang:** Methodology (equal); software (equal); writing – review and editing (equal). **Min Li:** Methodology (equal); software (equal); writing – review and editing (equal). **Guy Smagghe:** Writing – review and editing (equal). **RenHuai Dai:** Funding acquisition (equal); project administration (equal); supervision (equal); writing – review and editing (equal).

## FUNDING INFORMATION

National Natural Science Foundation of China (No. 32160119); the National Natural Science Foundation of China (No. 32000329); and the Program of Excellent Innovation Talents, Guizhou Province, China (No. 20206003‐2).

## CONFLICT OF INTEREST STATEMENT

All authors declare no conflict of interest.

## DATA AVAILABILITY STATEMENT

The complete mitochondrial genome of 26 Macropsini species in this study are available from the NCBI GenBank under the accession numbers listed in Table 1 (Section 2).

## Supporting information


Figure S1.

Table S1.

Table S2.
